# Clinical application of MR-Linac in tumor radiotherapy: a systematic review

**DOI:** 10.1186/s13014-023-02221-8

**Published:** 2023-03-14

**Authors:** Xin Liu, Zhenjiang Li, Yong Yin

**Affiliations:** 1 Department of Oncology, Affiliated Hospital of Southwest Medical University, Luzhou, 646000 China; 2 Department of Radiation Physics, Shandong Cancer Hospital and Institute, Shandong First Medical University and Shandong Academy of Medical Sciences, Jinan, 250117 China

**Keywords:** Radiotherapy, MR-Linac, MRgRT, Tumor, Cancer

## Abstract

Recent years have seen both a fresh knowledge of cancer and impressive advancements in its treatment. However, the clinical treatment paradigm of cancer is still difficult to implement in the twenty-first century due to the rise in its prevalence. Radiotherapy (RT) is a crucial component of cancer treatment that is helpful for almost all cancer types. The accuracy of RT dosage delivery is increasing as a result of the quick development of computer and imaging technology. The use of image-guided radiation (IGRT) has improved cancer outcomes and decreased toxicity. Online adaptive radiotherapy will be made possible by magnetic resonance imaging-guided radiotherapy (MRgRT) using a magnetic resonance linear accelerator (MR-Linac), which will enhance the visibility of malignancies. This review's objectives are to examine the benefits of MR-Linac as a treatment approach from the perspective of various cancer patients' prognoses and to suggest prospective development areas for additional study.

## Introduction

One crucial method of treating tumors is radiotherapy (RT). Statistics show that radiotherapy is required for more than 50% of cancer patients [1]. Therefore, the advancement of radiation will be crucial in enhancing patients' prognoses and minimizing adverse effects. Both volumetric modulated arc therapy (VMAT) and intensity-modulated radiotherapy (IMRT) are currently used in conventional radiotherapy techniques that achieve uniform radiation dose distribution. However, image guidance is required to achieve correct dose transmission and increase treatment accuracy [2]. Cone beam computed tomography (CBCT) is the most common image-guided radiation (IGRT) technique used in radiotherapy [3]. However, there are some drawbacks to CBCT imaging, including excessive scattering, poor image quality, ionizing radiation, and more [4, 5].

Magnetic resonance imaging (MRI) has the benefits of high soft tissue contrast [6–8] and no ionizing radiation compared to computerized tomography (CT) and CBCT, which can improve the accuracy of tumor target delineation and obtain biological information and functional data of tumor and normal tissues. MRI has developed into a crucial tool for identifying clinical targets and organs in danger. MR and linear accelerator have currently been coupled to create a magnetic resonance accelerator (MR-Linac) [8], a novel piece of radiation equipment that is directed by MR [9, 10]. In addition to offering superior target pictures than CBCT, MR can raise the target dose and improve the robustness [11] of adaptive radiotherapy to obtain a high tumor cure rate. According to several studies [12, 13], MR-guided radiation (MRgRT) is possible, effective, and considerably improves patient prognosis when used to treat tumors [14]. This article mostly reviews the development of MRgRT research in the management of various cancer types.

## Clinical practice

The most common cancers that are currently being treated with MR-Linac are listed in Table 1, together with information about the treatment, patient count, median follow-up period, overall survival rate, and advanced toxicity. There is a discussion of the thorough descriptions of several malignancies.

**Table 1:** Main types of cancer treated with MR-Linac

Tumor sites	Author	Time period	Reference	Treatment	No. of patients	Median follow-up (months)	Overall survival	Toxicity rates (≥ 3) (%)
Nasopharyngeal carcinoma	Fu S et al	4/2018–1/2020	[25]	70.4 Gy/32F	130	25	100% (2 years)	1.5
Lung tumor	Finazzi T et al	2016–2018	[41]	60 Gy/8F (n = 28), 55 Gy/5F (n = 23), 54 Gy/3F (n = 2), 60 Gy/12F (n = 1)	54	21.7	88.0% (1 years)	8
Breast cancer	Nachbar M et al	1/2019	[50]	40.05 Gy/15F	1	3	–	0
Pancreatic cancer	Chuong MD et al	2018–2021	[62]	50 Gy/5F	62	18.6	40% (2 years)	4.8
Cholangiocarcinoma	Luterstein E et al	5/2015–8/2017	[69]	40 Gy/5F	17	15.8	46.1% (2 years)	5.9
Prostatic cancer	Bruynzeel AME et al	10/2019–1/2020	[78]	35 Gy/5F	25	–	–	0

*F* fractions

## Head and neck tumors

As opposed to the chest, abdominal, and pelvic organs, which are more susceptible to motion errors, MR images have advantages in identifying brain tissue, muscle, and nerve. As a result, the target area can be defined using the MRI's superior soft tissue contrast [15, 16]. In a comparison of patients with retropharyngeal lymph node (RPLN) metastases, MRI was found to be more sensitive to detect lymph nodes than CT (74% vs. 65%) [17]. MRI was useful for evaluating dura mater, intracranial, and orbital invasion in nasopharyngeal cancer, and it considerably improved the detection rate of intracranial and pterygopalatine fossa invasion compared to CT [18, 19]. Particularly crucial for radiation for head and neck malignancies, MRI has a high rate of detection for distant metastases associated with poor prognosis. The size, shape, and number of targets [20], which correlate to the tumor stage and have a direct impact on the effectiveness of radiation, are the primary factors that determine how malignant a tumor is. By repeating MRI, the use of MR-Linac during radiotherapy can more clearly demonstrate the tumor response and target changes and enhance the therapeutic impact [21]. The frequency of persistent dysphagia in oropharyngeal cancer (OPC) patients treated with MRgRT fell by 11% compared to intensity-modulated radiation, and the average dosage to the parotid gland was reduced by 3.3 Gy [22], effectively protecting OARs and minimizing adverse effects. According to Ding et al., 50% of OPC patients who had MRgRT experienced complete remission [23, 24]. Another study found that patients with nasopharyngeal cancer (NPC) treated with MRgRT had a 2-year disease-free survival rate of 93.6%, compared to intensity-modulated radiotherapy's 87.5%. Acute toxicity did not differ significantly [25]. As a result, it is possible to apply MRgRT to head and neck malignancies [26, 27], which can not only successfully control the growth of tumors but also lessen the side effects that patients experience [28, 29]. The intra- and inter-fractional movement of head and neck tumors are, however, rather limited when compared to other lesions [30], therefore a proper benefit ratio analysis is crucial to gauge the effectiveness of the current and future treatments for MR-Linac in head and neck tumors.

## Chest tumors

Radiotherapy for chest malignancies is primarily influenced by respiratory exercise. Respiratory gating, abdominal control, and employing a CT simulator (4D-CT) to monitor the overall amplitude of the goal movement are the main techniques for controlling respiratory movement in different fractions [31]. The inability of 4D-CT to show intra- and inter-fraction anatomical changes of the target in real-time during treatment makes target definition more challenging. The error in the treatment process is increased for low-fractionated or ultra-low-fractionated stereotactic radiation (Stereotactic Body Radiation Therapy, SBRT), hence accuracy is a requirement for radiotherapy [32, 33]. The target coverage and OARs dose limitation can be greatly enhanced by utilizing the real-time gating technology of MR-Linac and SBRT, which allows for a more precise evaluation of intra- and inter-step changes in anatomy and function [34]. The accuracy of radiation can be considerably improved by MRgRT, which is more useful for chest cancers that are most impacted by respiratory movement. It can both shrink PTV and enhance dose [35]. The PTV delineated with gating and online adaptation on MR-Linac was found in a study of early peripheral non-small cell lung cancer (NSCLC) lesions to be 53.7% smaller than that based on 4DCT [36], demonstrating that MRgRT can not only lower the dose to normal tissue but also improve the accuracy of target coverage when compared to CT-guided radiotherapy. In 22 patients with lower lobe lung cancer, a retrospective analysis revealed that MRgRT was superior to VMAT in terms of target dosage uniformity and OARs dose limiting [37]. Chest malignancies respond well to MRgRT in terms of toxicity and side effect reduction, and the radiation dose to OARs is also greatly reduced [38]. The likelihood of toxic events will be considerably raised when radiotherapy for a chest tumor is administered because the bronchus, esophagus, heart, and main blood vessels, as well as the brachial plexus, spinal cord, phrenic nerve, and recurrent laryngeal nerve, are nearby structures [39]. According to Machtay et al.’s meta-analysis report, the biologically effective dose (BED) has a 4% survival advantage with each increment of 1 Gy [40], Other research revealed that the MR-Linac treatment for lung cancer had 12-month local control rates of 95.6%, overall survival rates of 88.0%, and disease-free survival rates of 63.6%. There were no toxicities rated 4–5 found [41]. It is indicated that MR-Linac is a potent tool for treating locally progressed diseases that can safely raise the effective dose.

When used in breast cancer radiotherapy, MR-Linac is superior to a traditional accelerator in terms of displaying breast tissue, targets, and postoperative changes [42, 43], clearly displaying carcinoma in situ, reducing radiation exposure, quickly adjusting the daily schedule, and offering patients individualized treatment plans [44]. However, the Lorentz force of the magnetic field will deflect the secondary electrons, alter the distribution of the tissue dose, increase the dose in the skin and chest wall of the radiation field, cause adverse reactions like skin radioactive ulcers, and lower the quality of life for patients [45–47]. Studies have demonstrated that MRgRT is more efficient at irradiating breast cancer before surgery because it uses the signal difference between the tumor and the surrounding glandular tissue to more precisely define the target [48, 49]. A 1.5 T Elekta MR-Linac investigation of adjuvant partial breast irradiation (PBI) revealed only grade 1 toxicity in the breast [50]. Another study contrasted the MR-Linac and VMAT's differing dosimetric properties. The outcomes demonstrated that MR was more successful in defending the ipsilateral breast and chest wall [51]. There are, however, not many reports on the use of MR accelerators in radiotherapy for breast cancer. The effect of the electron cyclotron effect in breast cancer radiation should be taken into consideration by MR accelerators due to the influence of magnetic fields on electrons, and steps should be taken to avoid extra side effects.

## Abdominal tumors

Pancreatic cancer is difficult to detect early and has a low survival rate [52]. Although surgical resection is a crucial component of early pancreatic cancer treatment, more than 80% of patients already have advanced pancreatic cancer at the time of their diagnosis [53]. One of the treatments for pancreatic cancer that cannot be operated on is neoadjuvant radiation. But the pancreas inhabits a very intricate anatomical setting, and its vital organs move considerably when breathing. The common bile duct and the duodenum are both close to the head of the pancreas. The jejunum, stomach, kidney, spleen, and major blood arteries are close to the pancreas' neck, body, and tail. Patients' intestines and stomachs may react poorly to high-dose radiation [54]. To prevent severe toxic effects, the acceptable dose of the surrounding tissues and organs should be taken into account when planning and administering radiation. The MR-Linac is appropriate for dose enhancement of tumor targets because of its high picture quality and online adaptive planning capability [55], especially for tumors with significant abdominal and pelvic mobility [56]. The adaptive plan may be noticed in the MR imaging of the MR-Linac, which not only optimizes the target coverage area but also successfully lowers the dose of OARs [57, 58]. In a study using MR-Linac, the toxicity was found to be significantly reduced in patients with locally advanced pancreatic cancer. Over the median follow-up of 13 months, only one incident of grade 1 toxicity and no advanced toxicity were recorded, which was a significant reduction from the usual criterion [59]. Since other retrospective investigations have produced comparable findings, using adaptive radiation can reduce adverse effects and enhance patient prognosis [60, 61]. Chuong MD et al. discovered that the 2-year local control rate and 2-year OS rate of patients getting high-dose treatment in MRgRT were, respectively, 87.8% and 45.5% via a median follow-up of 17 months for pancreatic cancer patients who received radiotherapy. The 2-year overall survival rate was statistically higher compared to other cohorts [62]. MRgRT is utilized to modify the radiation plan in real-time for more precise dose distribution to lower toxicity, increase efficacy, and lower the likelihood of recurrence in the treatment of pancreatic cancer.

The similarity of the electron density in the abdominal cavity makes it more challenging to use CBCT for radiation planning because of the movement of the diaphragm and changes in organ volume during the course of liver cancer treatment [63, 64]. MRI has excellent soft tissue resolution [65], which makes it useful for locating tumor targets and delivering more precise and effective care. The target dose might be impacted by the mobility of OARs in the abdominal cavity. Through real-time viewing, MRgRT can improve the flaws in present abdominal radiation [66, 67]. MR imaging meets the standards for dose transfer precision and provides a better display effect on 77% of abdominal tumor targets and OARs than normal CBCT imaging [68]. A patient with acute grade 3 gastrointestinal toxicity was detected in the trial of 17 patients with unresectable locally advanced cholangiocarcinoma treated with MRgRT, which was much better than the 10–26% grade 3 gastrointestinal toxicity shown by meta-analysis [69, 70]. MRgRT has dosimetric advantages in target coverage and OAR protection for primary or metastatic liver lesions [71, 72]. All things considered, MRgRT is safe and practicable for patients with abdominal malignancies.

## Genitourinary system tumors

External radiation therapy for prostate cancer commonly causes dysuria, frequent urination, diarrhea, and rectal urgency among its acute adverse effects. Urinary stricture, cystitis, proctitis, and sexual dysfunction are long-term adverse effects [73, 74]. Therefore, the key to raising patients' quality of life following radiotherapy is to lessen the toxicity and side effects of prostate cancer. When treating prostate cancer, MR-Linac can adjust to structural changes as the disease progresses and reduce movement-related errors following intestine and bladder filling [75, 76]. According to a study, prior to actual radiation treatment, the pelvic organs would shift as a result of the course of image acquisition and planning adjustments, changing the PTV. As a result, it is required to modify the radiotherapy plan to take into account the altered anatomical structure. Therefore, an essential method of ensuring the use of an exact dose to decrease toxicity during radiation is the motion monitoring mechanism of the MR-Linac [77]. The incidence of grade 2 or higher acute genitourinary toxicity was found to be 23.8% in a prospective study [78] of 101 patients with moderate or high risk localized prostate cancer treated with MRgRT. This is significantly less frequent than the likelihood of toxic reactions when using conventional accelerators [79, 80]. The findings from a different study were comparable. Early prostate cancer patients experienced 5% grade 2 gastrointestinal toxicity, but no grade 3 toxicity [81]. When compared to CT-guided radiotherapy, Ma TM et al. discovered that MR-guided radiotherapy for prostate cancer dramatically reduced acute genitourinary toxicity and improved urine and intestinal function [82]. These findings support the notion that MRgRT can be used to treat prostate cancer more effectively clinically [83]. We can lower the dose of OARs and further reduce side effects for patients by responding to the changes in intestinal and bladder position by using the changes of target and OARs observed on daily MRI and the online adaptive therapy offered by MR-Linac [84]. Overall, radiation advantages can be increased by safe, efficient treatment with a low hazardous burden. Daily adaptive Mr-guided SBRT is a viable and accurate treatment strategy for prostate cancer [85].

MRI offers superior soft tissue contrast to conventional imaging techniques [86] and has taken over as the primary way of staging cervical cancer [87]. The use of MRI in extracorporeal radiation (EBRT) for gynecological malignancies can prevent missing beams in the target area [88]. Numerous experimental findings have demonstrated that IGRT can enhance patient prognosis, increase survival rate, and decrease therapeutic toxicity [89–92]. In order to create a breakthrough in the treatment of gynecological malignant tumors, MRgRT combines the benefits of both approaches. The goal of MRgRT is to reduce therapeutic toxicity and optimize treatment by performing online adaptive radiotherapy based on the anatomical structure of the day, shrinking the scope of GTV, monitoring anatomical changes during treatment, and increasing the target dosage. Compared to CBCT-guided radiation, the daily online adaptive plan employing MR-Linac can increase the target dose [93]. In their investigation, Cree An et al. discovered that the combined therapy had a reported incidence of adverse events (grade 3) as low as 3.7% and a 5-year survival rate of 78.5% for 1322 patients with endometrial cancer [94]. As a result, MRgRT is secure and successful in treating gynecological cancers.

## Prospect

The clinical use of MRI in conjunction with radiotherapy is discussed in this study for the treatment of cancer. Radiation oncology is using MR-Linac as one of its key instruments. The use of MRgRT, which combines the imaging benefits of MRI with RT and has significant potential for treating tumors, advances the field of radiation. Large amounts of imaging data acquired during MRgRT can be used to extract a variety of data sources, which can then be coupled with machine learning and artificial intelligence to incorporate new MRI features [95, 96]. The development of radiation dosage individualization and re-irradiation can be accelerated by the use of MRI in the future [97]. It can also be used to measure patients' risk classification and prognosis, enhance patient prognosis, lower complications, and boost overall survival. It can qualitatively enhance the therapeutic impact on cancer patients [98].

Given the clinical and technical issues raised, MRgRT's future research should concentrate on advancing imaging technologies and bolstering the high-dimensional quantization of pictures. Biomarkers of pictures are utilized to direct the intensification or de-enhancement of radiation, systemic, and surgical techniques in regions like the abdomen and pelvis. To get better results, it would be ideal if this study used a multicenter approach to general data analysis. Clinical investigations should be carried out following pre-treatment evaluation in order to make sure that the edge of the target that should be included in the conventional PTV will not be missed, even though MRgRT can increase treatment accuracy and decrease PTV. Since PET/MRI imaging is still in its infancy, it is possible to examine the potential synergy of the two in the use of MRgRT technology by using better imaging techniques. Radiomics can be used to enhance the clinical outcome of patients receiving MRgRT by enhancing image guidance methods and utilizing functional imaging biomarkers [99].

Artificial intelligence (AI) advancement has altered our lives in recent years. With the help of AI, radiomics has benefited from significant advancements, including the ability to automatically draw regions of interest (ROI), the introduction of neural networks (NN) that can directly infer image features from ROI, and the advancement of machine learning (ML) and deep learning (DL) algorithms [100] to streamline predictive models to inform treatment decisions [101, 102]. The widespread use of AI in RT has enormous promise and can significantly advance numerous processes, from diagnosis to treatment [103, 104]. The effectiveness of the MR-Linac online adaptive program is hampered by the lengthy treatment times, hence there is an urgent need for manual process automation to reduce the treatment times [105, 106]. The field of RT is undergoing revolutionary changes as a result of the development of AI. To reach the goal of a reduced treatment time, it offers a DL-based system that can produce electronic density (ED) maps one by one voxel [107, 108]. In order to increase the precision of dose distribution, AI is expected to work in collaboration with MR-Linac to make a significant contribution to the study of dosage augmentation. Nearly 90% of the cases in the study of 203 individuals with nasopharyngeal cancer utilizing the DL approach didn't require expert manual re-editing [109]. Chen et al. processed the magnetic resonance images of 20 patients with abdominal tumors using a type of automated deep learning-based abdominal multi-organ segmentation (ALAMO). The outcomes demonstrate that most organs can be segmented accurately, and the therapy takes around 18 s, which is completely compatible with online operation [110]. All things considered, the incorporation of AI may considerably raise the level of MRgRT treatment over the course of the next few years and be crucial to the development of customized cancer treatment. However, the proper use of AI in radiation oncology is also a significant problem, and all participants must learn new procedures while receiving irradiation under the supervision of experts [111, 112].

As of right now, this technology is still in its infancy, the full potential of AI has not yet been realized, the clinical data are not yet mature, and there are still certain issues, including the standard of MRgRT workflow, that need to be further resolved by academics. In the future, we'll investigate the true potential of online adaptive radiotherapy, gradually advance the clinical use of MR-Linac, demonstrate the viability of MR-based radiotherapy, and identify the patients who will profit from it the most.

## Conclusion

In conclusion, MRgRT has played a significant role in the evolution of radiotherapy. The clinical outcomes of MR-guided radiotherapy will be closely monitored throughout the coming years, which will advance the new knowledge of tumor therapy.

## References

[1] Atun R, Jaffray DA, Barton MB (2015). Expanding global access to radiotherapy. Lancet Oncol.

[2] Srinivasan K, Mohammadi M, Shepherd J (2014). Applications of Linac-mounted kilovoltage cone-beam computed tomography in modern radiation therapy: a review. Pol J Radiol.

[3] Jaffray DA, Siewerdsen JH (2000). Cone-beam computed tomography with a flat-panel imager: initial performance characterization. Med Phys.

[4] Higgins J, Bezjak A, Franks K (2009). Comparison of spine, carina, and tumor as registration landmarks for volumetric image-guided lung radiotherapy. Int J Radiat Oncol Biol Phys.

[5] Yadav P, Chang SX, Cheng CW, DesRosiers CM, Mitra RK, Das IJ (2022). Dosimetric evaluation of high-Z inhomogeneity used for hip prosthesis: a multi-institutional collaborative study. Phys Med.

[6] Wang H, Chandarana H, Block KT, Vahle T, Fenchel M, Das IJ (2017). Dosimetric evaluation of synthetic CT for magnetic resonance-only based radiotherapy planning of lung cancer. Radiat Oncol.

[7] Wang H, Du K, Qu J, Chandarana H, Das IJ (2018). Dosimetric evaluation of magnetic resonance-generated synthetic CT for radiation treatment of rectal cancer. PLoS ONE.

[8] Farjam R, Tyagi N, Deasy JO, Hunt MA (2019). Dosimetric evaluation of an atlas-based synthetic CT generation approach for MR-only radiotherapy of pelvis anatomy. J Appl Clin Med Phys.

[9] Kerkmeijer LG, Fuller CD, Verkooijen HM (2016). The MRI-Linear accelerator consortium: evidence-based clinical introduction of an innovation in radiation oncology connecting researchers, methodology, data collection, quality assurance, and technical development. Front Oncol.

[10] Roberts DA, Sandin C, Vesanen PT (2021). Machine QA for the Elekta Unity system: a report from the Elekta MR-Linac consortium. Med Phys.

[11] Lattanzi J, McNeeley S, Pinover W (1999). A comparison of daily CT localization to a daily ultrasound-based system in prostate cancer. Int J Radiat Oncol Biol Phys.

[12] Randall JW, Rammohan N, Das IJ, Yadav P (2022). Towards accurate and precise image-guided radiotherapy: clinical applications of the MR-Linac. J Clin Med.

[13] Hehakaya C, van der Voort van Zyp JRN, Vanneste BGL (2021). Early health economic analysis of 1.5 T MRI-guided radiotherapy for localized prostate cancer: decision analytic modelling. Radiother Oncol..

[14] Henke LE, Contreras JA, Green OL (2018). Magnetic resonance image-guided radiotherapy (MRIgRT): a 4.5-year clinical experience. Clin Oncol.

[15] Jensen K, Friborg J, Hansen CR (2020). The Danish Head and Neck Cancer Group (DAHANCA) 2020 radiotherapy guidelines. Radiother Oncol.

[16] Jensen K, Al-Farra G, Dejanovic D (2021). Imaging for target delineation in head and neck cancer radiotherapy. Semin Nucl Med.

[17] Kim JH, Choi KY, Lee SH (2020). The value of CT, MRI, and PET-CT in detecting retropharyngeal lymph node metastasis of head and neck squamous cell carcinoma. BMC Med Imaging.

[18] Chung NN, Ting LL, Hsu WC, Lui LT, Wang PM (2004). Impact of magnetic resonance imaging versus CT on nasopharyngeal carcinoma: primary tumor target delineation for radiotherapy. Head Neck.

[19] Dirix P, Haustermans K, Vandecaveye V (2014). The value of magnetic resonance imaging for radiotherapy planning. Semin Radiat Oncol.

[20] Forghani R, Yu E, Levental M, Som PM, Curtin HD (2015). Imaging evaluation of lymphadenopathy and patterns of lymph node spread in head and neck cancer. Expert Rev Anticancer Ther.

[21] Ahmed M, Schmidt M, Sohaib A (2010). The value of magnetic resonance imaging in target volume delineation of base of tongue tumours—a study using flexible surface coils. Radiother Oncol.

[22] Mohamed ASR, Bahig H, Aristophanous M (2018). Prospective *in silico* study of the feasibility and dosimetric advantages of MRI-guided dose adaptation for human papillomavirus positive oropharyngeal cancer patients compared with standard IMRT. Clin Transl Radiat Oncol.

[23] Subesinghe M, Scarsbrook AF, Sourbron S (2015). Alterations in anatomic and functional imaging parameters with repeated FDG PET-CT and MRI during radiotherapy for head and neck cancer: a pilot study. BMC Cancer.

[24] Ding Y, Hazle JD, Mohamed AS (2015). Intravoxel incoherent motion imaging kinetics during chemoradiotherapy for human papillomavirus-associated squamous cell carcinoma of the oropharynx: preliminary results from a prospective pilot study. NMR Biomed.

[25] Fu S, Li Y, Han Y (2022). Diffusion-weighted magnetic resonance imaging-guided dose painting in patients with locoregionally advanced nasopharyngeal carcinoma treated with induction chemotherapy plus concurrent chemoradiotherapy: a randomized, controlled clinical trial. Int J Radiat Oncol Biol Phys.

[26] McDonald BA, Vedam S, Yang J (2021). Initial feasibility and clinical implementation of daily MR-guided adaptive head and neck cancer radiation therapy on a 1.5T MR-Linac system: prospective R-IDEAL 2a/2b systematic clinical evaluation of technical innovation. Int J Radiat Oncol Biol Phys..

[27] de Mol van Otterloo SR, Christodouleas JP, Blezer ELA (2021). Patterns of care, tolerability, and safety of the first cohort of patients treated on a novel high-field MR-Linac within the MOMENTUM study: initial results from a prospective multi-institutional registry. Int J Radiat Oncol Biol Phys..

[28] Chen AM, Cao M, Hsu S (2017). Magnetic resonance imaging guided reirradiation of recurrent and second primary head and neck cancer. Adv Radiat Oncol.

[29] Chen AM, Hsu S, Lamb J (2018). MRI-guided radiotherapy for head and neck cancer: initial clinical experience. Clin Transl Oncol.

[30] Bruijnen T, Stemkens B, Terhaard CHJ, Lagendijk JJW, Raaijmakers CPJ, Tijssen RHN (2019). Intrafraction motion quantification and planning target volume margin determination of head-and-neck tumors using cine magnetic resonance imaging. Radiother Oncol.

[31] Brandner ED, Chetty IJ, Giaddui TG, Xiao Y, Huq MS (2017). Motion management strategies and technical issues associated with stereotactic body radiotherapy of thoracic and upper abdominal tumors: a review from NRG oncology. Med Phys.

[32] Videtic GMM, Donington J, Giuliani M (2017). Stereotactic body radiation therapy for early-stage non-small cell lung cancer: executive summary of an ASTRO EVIDENCE-BASED GUIDEline. Pract Radiat Oncol.

[33] Hingorani M, Colley WP, Dixit S, Beavis AM (2012). Hypofractionated radiotherapy for glioblastoma: strategy for poor-risk patients or hope for the future?. Br J Radiol.

[34] Henke L, Kashani R, Yang D (2016). Simulated online adaptive magnetic resonance-guided stereotactic body radiation therapy for the treatment of oligometastatic disease of the abdomen and central thorax: characterization of potential advantages. Int J Radiat Oncol Biol Phys.

[35] Shirato H, Seppenwoolde Y, Kitamura K, Onimura R, Shimizu S (2004). Intrafractional tumor motion: lung and liver. Semin Radiat Oncol.

[36] Finazzi T, Palacios MA, Haasbeek CJA (2020). Stereotactic MR-guided adaptive radiation therapy for peripheral lung tumors. Radiother Oncol.

[37] Park JM, Wu HG, Kim HJ, Choi CH, Kim JI (2019). Comparison of treatment plans between IMRT with MR-Linac and VMAT for lung SABR. Radiat Oncol.

[38] Padgett KR, Simpson GN, Llorente R, Samuels MA, Dogan N (2018). Feasibility of adaptive MR-guided stereotactic body radiotherapy (SBRT) of lung tumors. Cureus..

[39] Timmerman R, McGarry R, Yiannoutsos C (2006). Excessive toxicity when treating central tumors in a phase II study of stereotactic body radiation therapy for medically inoperable early-stage lung cancer. J Clin Oncol.

[40] Machtay M, Bae K, Movsas B (2012). Higher biologically effective dose of radiotherapy is associated with improved outcomes for locally advanced non-small cell lung carcinoma treated with chemoradiation: an analysis of the Radiation Therapy Oncology Group. Int J Radiat Oncol Biol Phys.

[41] Finazzi T, Haasbeek CJA, Spoelstra FOB (2020). Clinical outcomes of stereotactic MR-guided adaptive radiation therapy for high-risk lung tumors. Int J Radiat Oncol Biol Phys.

[42] Groot Koerkamp ML, Vasmel JE, Russell NS (2020). Optimizing MR-guided radiotherapy for breast cancer patients. Front Oncol.

[43] Khoo VS, Dearnaley DP, Finnigan DJ, Padhani A, Tanner SF, Leach MO (1997). Magnetic resonance imaging (MRI): considerations and applications in radiotherapy treatment planning. Radiother Oncol.

[44] Glide-Hurst CK, Paulson ES, McGee K (2021). Task group 284 report: magnetic resonance imaging simulation in radiotherapy: considerations for clinical implementation, optimization, and quality assurance. Med Phys.

[45] van Heijst TC, den Hartogh MD, Lagendijk JJ, van den Bongard HJ, van Asselen B (2013). MR-guided breast radiotherapy: feasibility and magnetic-field impact on skin dose. Phys Med Biol.

[46] Esmaeeli AD, Mahdavi SR, Pouladian M, Monfared AS, Bagheri S (2014). Improvement of dose distribution in breast radiotherapy using a reversible transverse magnetic field Linac-MR unit. Med Phys..

[47] Chen X, Prior P, Chen GP, Schultz CJ, Li XA (2016). Technical note: Dose effects of 1.5 T transverse magnetic field on tissue interfaces in MRI-guided radiotherapy. Med Phys..

[48] Horton JK, Blitzblau RC, Yoo S (2015). Preoperative single-fraction partial breast radiation therapy: a novel phase 1, dose-escalation protocol with radiation response biomarkers. Int J Radiat Oncol Biol Phys.

[49] den Hartogh MD, Philippens ME, van Dam IE (2014). MRI and CT imaging for preoperative target volume delineation in breast-conserving therapy. Radiat Oncol.

[50] Nachbar M, Mönnich D, Boeke S (2020). Partial breast irradiation with the 1.5 T MR-Linac: first patient treatment and analysis of electron return and stream effects. Radiother Oncol..

[51] Musunuru HB, Yadav P, Olson SJ, Anderson BM (2021). Improved ipsilateral breast and chest wall sparing with MR-guided 3-fraction accelerated partial breast irradiation: a dosimetric study comparing MR-Linac and CT-Linac plans. Adv Radiat Oncol..

[52] Joshi SS, Badgwell BD (2021). Current treatment and recent progress in gastric cancer. CA Cancer J Clin.

[53] Gillen S, Schuster T, Meyer Zum Büschenfelde C, Friess H, Kleeff J (2010). Preoperative/neoadjuvant therapy in pancreatic cancer: a systematic review and meta-analysis of response and resection percentages. PLoS Med..

[54] Chang DT, Schellenberg D, Shen J (2009). Stereotactic radiotherapy for unresectable adenocarcinoma of the pancreas. Cancer.

[55] Hawranko R, Sohn JJ, Neiderer K (2022). Investigation of isotoxic dose escalation and plan quality with TDABC analysis on a 0.35 T MR-Linac (MRL) system in ablative 5-fraction stereotactic magnetic resonance-guided radiation therapy (MRgRT) for primary pancreatic cancer. J Clin Med..

[56] Hassanzadeh C, Rudra S, Bommireddy A (2020). Ablative five-fraction stereotactic body radiation therapy for inoperable pancreatic cancer using online MR-guided adaptation. Adv Radiat Oncol..

[57] Chin S, Eccles CL, McWilliam A (2020). Magnetic resonance-guided radiation therapy: a review. J Med Imaging Radiat Oncol.

[58] Placidi L, Romano A, Chiloiro G (2020). On-line adaptive MR guided radiotherapy for locally advanced pancreatic cancer: clinical and dosimetric considerations. Tech Innov Patient Support Radiat Oncol.

[59] Pollom EL, Alagappan M, von Eyben R (2014). Single- versus multifraction stereotactic body radiation therapy for pancreatic adenocarcinoma: outcomes and toxicity. Int J Radiat Oncol Biol Phys.

[60] Chuong MD, Bryant J, Mittauer KE (2021). Ablative 5-fraction stereotactic magnetic resonance-guided radiation therapy with on-table adaptive replanning and elective nodal irradiation for inoperable pancreas cancer [published correction appears in Pract Radiat Oncol. 2021 May-Jun;11(3):e354]. Pract Radiat Oncol..

[61] Rudra S, Jiang N, Rosenberg SA (2019). Using adaptive magnetic resonance image-guided radiation therapy for treatment of inoperable pancreatic cancer. Cancer Med.

[62] Chuong MD, Herrera R, Kaiser A (2022). Induction chemotherapy and ablative stereotactic magnetic resonance image-guided adaptive radiation therapy for inoperable pancreas cancer. Front Oncol..

[63] Hall WA, Paulson E, Li XA (2022). Magnetic resonance linear accelerator technology and adaptive radiation therapy: an overview for clinicians. CA Cancer J Clin.

[64] Otazo R, Lambin P, Pignol JP (2021). MRI-guided radiation therapy: an emerging paradigm in adaptive radiation oncology. Radiology.

[65] Yadav P, Kuczmarska-Haas A, Musunuru HB (2021). Evaluating dose constraints for radiation induced liver damage following magnetic resonance image guided stereotactic body radiotherapy. Phys Imaging Radiat Oncol.

[66] Paganelli C, Whelan B, Peroni M (2018). MRI-guidance for motion management in external beam radiotherapy: current status and future challenges. Phys Med Biol..

[67] Wolthaus JW, Sonke JJ, van Herk M (2008). Comparison of different strategies to use four-dimensional computed tomography in treatment planning for lung cancer patients. Int J Radiat Oncol Biol Phys.

[68] Noel CE, Parikh PJ, Spencer CR (2015). Comparison of onboard low-field magnetic resonance imaging versus onboard computed tomography for anatomy visualization in radiotherapy. Acta Oncol.

[69] Luterstein E, Cao M, Lamb JM (2019). Clinical outcomes using magnetic resonance-guided stereotactic body radiation therapy in patients with locally advanced cholangiocarcinoma. Adv Radiat Oncol.

[70] Lee J, Yoon WS, Koom WS, Rim CH (2019). Efficacy of stereotactic body radiotherapy for unresectable or recurrent cholangiocarcinoma: a meta-analysis and systematic review. Wirksamkeit der stereotaktischen Strahlentherapie bei nichtresektablem oder rezidivierendem Cholangiokarzinom: eine Metaanalyse und systematische Übersicht. Strahlenther Onkol..

[71] Padgett KR, Simpson G, Asher D, Portelance L, Bossart E, Dogan N (2020). Assessment of online adaptive MR-guided stereotactic body radiotherapy of liver cancers. Phys Med.

[72] Mayinger M, Ludwig R, Christ SM (2021). Benefit of replanning in MR-guided online adaptive radiation therapy in the treatment of liver metastasis. Radiat Oncol.

[73] Katz AJ, Kang J (2014). Quality of life and toxicity after SBRT for organ-confined prostate cancer, a 7-year study. Front Oncol.

[74] Teunissen FR, van der Voort van Zyp JRN, Verkooijen HM, Wortel RC (2022). Neurovascular-sparing MR-guided adaptive radiotherapy in prostate cancer; defining the potential population for erectile function-sparing treatment. J Sex Med..

[75] Jmour O, Benna M, Champagnol P (2020). CBCT evaluation of inter- and intra-fraction motions during prostate stereotactic body radiotherapy: a technical note. Radiat Oncol.

[76] Sheng Y, Li T, Lee WR, Yin FF, Wu QJ (2017). Exploring the margin recipe for online adaptive radiation therapy for intermediate-risk prostate cancer: an intrafractional seminal vesicles motion analysis. Int J Radiat Oncol Biol Phys.

[77] Mannerberg A, Persson E, Jonsson J (2020). Dosimetric effects of adaptive prostate cancer radiotherapy in an MR-Linac workflow. Radiat Oncol.

[78] Bruynzeel AME, Tetar SU, Oei SS (2019). A prospective single-arm phase 2 study of stereotactic magnetic resonance guided adaptive radiation therapy for prostate cancer: early toxicity results. Int J Radiat Oncol Biol Phys.

[79] Incrocci L, Wortel RC, Alemayehu WG (2016). Hypofractionated versus conventionally fractionated radiotherapy for patients with localised prostate cancer (HYPRO): final efficacy results from a randomised, multicentre, open-label, phase 3 trial. Lancet Oncol.

[80] Dearnaley D, Syndikus I, Mossop H (2016). Conventional versus hypofractionated high-dose intensity-modulated radiotherapy for prostate cancer: 5-year outcomes of the randomised, non-inferiority, phase 3 CHHiP trial [published correction appears in Lancet Oncol. 2016 Aug;17 (8):e321]. Lancet Oncol..

[81] Alongi F, Rigo M, Figlia V (2020). 1.5 T MR-guided and daily adapted SBRT for prostate cancer: feasibility, preliminary clinical tolerability, quality of life and patient-reported outcomes during treatment. Radiat Oncol..

[82] Ma TM, Lamb JM, Casado M (2021). Magnetic resonance imaging-guided stereotactic body radiotherapy for prostate cancer (mirage): a phase iii randomized trial. BMC Cancer.

[83] Mazzola R, Figlia V, Rigo M (2020). Feasibility and safety of 1.5 T MR-guided and daily adapted abdominal-pelvic SBRT for elderly cancer patients: geriatric assessment tools and preliminary patient-reported outcomes. J Cancer Res Clin Oncol..

[84] Hoegen P, Spindeldreier CK, Buchele C (2021). Magnetresonanzgeführte Strahlentherapie : Beginn einer neuen Ära in der Radioonkologie? [Magnetic-resonance-guided radiotherapy: the beginning of a new era in radiation oncology?]. Radiologe.

[85] Nicosia L, Sicignano G, Rigo M (2021). Daily dosimetric variation between image-guided volumetric modulated arc radiotherapy and MR-guided daily adaptive radiotherapy for prostate cancer stereotactic body radiotherapy. Acta Oncol.

[86] Scoutt LM, McCarthy SM (1990). Applications of magnetic resonance imaging to gynecology. Top Magn Reson Imaging.

[87] Manganaro L, Lakhman Y, Bharwani N (2021). Staging, recurrence and follow-up of uterine cervical cancer using MRI: updated guidelines of the European Society of Urogenital Radiology after revised FIGO staging [published correction appears in Eur Radiol. 2021 Jun 17]. Eur Radiol..

[88] Russell AH, Anderson M, Walter J, Kinney W, Smith L, Scudder S (1992). The integration of computed tomography and magnetic resonance imaging in treatment planning for gynecologic cancer. Clin Obstet Gynecol.

[89] Tanderup K, Lindegaard JC, Kirisits C (2016). Image guided adaptive brachytherapy in cervix cancer: a new paradigm changing clinical practice and outcome. Radiother Oncol.

[90] Grover S, Harkenrider MM, Cho LP (2016). Image guided cervical brachytherapy: 2014 survey of the American Brachytherapy Society. Int J Radiat Oncol Biol Phys.

[91] Sturdza A, Pötter R, Fokdal LU (2016). Image guided brachytherapy in locally advanced cervical cancer: improved pelvic control and survival in RetroEMBRACE, a multicenter cohort study. Radiother Oncol.

[92] Pötter R, Tanderup K, Kirisits C (2018). The EMBRACE II study: the outcome and prospect of two decades of evolution within the GEC-ESTRO GYN working group and the EMBRACE studies. Clin Transl Radiat Oncol.

[93] Facondo G, Vullo G, DE Sanctis V, et al. Stereotactic body radiation therapy boost in patients with cervical cancer ineligible for brachytherapy. Cancer Diagn Progn. 2021;1(2):53–60. 10.21873/cdp.10008. 10.21873/cdp.10008 PMC8962763 35403131

[94] Cree A, Livsey J, Barraclough L (2018). The potential value of MRI in external-beam radiotherapy for cervical cancer. Clin Oncol (R Coll Radiol).

[95] Volpe S, Pepa M, Zaffaroni M (2021). Machine learning for head and neck cancer: a safe bet?—A clinically oriented systematic review for the radiation oncologist. Front Oncol..

[96] Werth K, Ledbetter L (2020). Artificial intelligence in head and neck imaging: a glimpse into the future. Neuroimaging Clin N Am.

[97] He J, Ren J, Niu G (2022). Multiparametric MR radiomics in brain glioma: models comparation to predict biomarker status. BMC Med Imaging.

[98] Yang Y, Cao M, Sheng K (2016). Longitudinal diffusion MRI for treatment response assessment: preliminary experience using an MRI-guided tri-cobalt 60 radiotherapy system. Med Phys.

[99] Boldrini L, Cusumano D, Chiloiro G (2019). Delta radiomics for rectal cancer response prediction with hybrid 0.35 T magnetic resonance-guided radiotherapy (MRgRT): a hypothesis-generating study for an innovative personalized medicine approach. Radiol Med..

[100] Forghani R, Savadjiev P, Chatterjee A, Muthukrishnan N, Reinhold C, Forghani B (2019). Radiomics and artificial intelligence for biomarker and prediction model development in oncology. Comput Struct Biotechnol J.

[101] Kulkarni S, Seneviratne N, Baig MS, Khan AHA (2020). Artificial intelligence in medicine: where are we now?. Acad Radiol.

[102] Lambin P, van Stiphout RG, Starmans MH (2013). Predicting outcomes in radiation oncology–multifactorial decision support systems. Nat Rev Clin Oncol.

[103] Francolini G, Desideri I, Stocchi G (2020). Artificial Intelligence in radiotherapy: state of the art and future directions. Med Oncol.

[104] Vandewinckele L, Claessens M, Dinkla A (2020). Overview of artificial intelligence-based applications in radiotherapy: recommendations for implementation and quality assurance. Radiother Oncol.

[105] Güngör G, Serbez İ, Temur B (2021). Time analysis of online adaptive magnetic resonance-guided radiation therapy workflow according to anatomical sites. Pract Radiat Oncol.

[106] Placidi L, Cusumano D, Boldrini L (2020). Quantitative analysis of MRI-guided radiotherapy treatment process time for tumor real-time gating efficiency. J Appl Clin Med Phys.

[107] Fu Y, Mazur TR, Wu X (2018). A novel MRI segmentation method using CNN-based correction network for MRI-guided adaptive radiotherapy. Med Phys.

[108] Wang J, Lu J, Qin G (2018). Technical note: A deep learning-based autosegmentation of rectal tumors in MR images. Med Phys.

[109] Lin L, Dou Q, Jin YM (2019). Deep learning for automated contouring of primary tumor volumes by MRI for nasopharyngeal carcinoma. Radiology.

[110] Chen Y, Ruan D, Xiao J (2020). Fully automated multiorgan segmentation in abdominal magnetic resonance imaging with deep neural networks. Med Phys.

[111] Fiorino C, Jeraj R, Clark CH (2020). Grand challenges for medical physics in radiation oncology. Radiother Oncol.

[112] Thompson RF, Valdes G, Fuller CD (2018). Artificial intelligence in radiation oncology: a specialty-wide disruptive transformation?. Radiother Oncol.

